# Calculation method of impingement depth and multiple objective optimizations of nozzle layout for aviation gears considering windage

**DOI:** 10.1038/s41598-025-01145-5

**Published:** 2025-05-17

**Authors:** Linlin Li, Yuanjun Ye, Yuzhong Zhang, Kai Zhao

**Affiliations:** 1 Xi’an Aeronautical Polytechnic Institute, Xi’an, 710089 China; 2 Northwestern Polytechnical University, Xi’an, 710072 China

**Keywords:** Aviation gear, Oil jet lubrication, Analysis model, Windage impingement depth, Multi-objective optimization, Aerospace engineering, Mechanical engineering

## Abstract

As the rotating speed of aviation gears becomes higher and higher, the influence of windage resistance on jet streamline must be considered in lubrication system design. To provide theoretical guidance for nozzle layout, an analytical model to calculate the windage impingement depth of gears is proposed. To obtain the best oil jet lubrication effect, the multi-objective optimization design method of oil jet streamline layout parameters with the largest impingement depth and the smallest impingement depth difference between two gears was proposed. CFD simulation analysis of flow field around gears are conducted to study the influence of jet nozzle layout parameters on the oil distribution and oil pressure. The results show that: the modulus and transmission ratio mainly affect the value range of oil jet streamline offset, and the correlation is positive. With the increase of inclination angle and initial offset, the impingement depth of the two gears is negatively correlated. The optimized oil jet streamline layout parameters of the case in the article are *S* = 0.53 mm, *β* = 4.6°, and the impingement depth difference between the two wheels of the gear pair after optimization is reduced by more than 50.3%. Calculation results are consistent with CFD, which validates the effectiveness and accuracy of the model. This research provides a design theory and method for the selection of oil lubrication parameters for aviation high-speed and heavy-load aviation gears.

## Introduction

The aviation gear pair mainly adopt the out-of-mesh oil jet lubrication, when working under high-speed conditions and high-power transmission^1,2^. However, when the lubricating oil and air are mixed around the gear to form oil-gas two-phase flow, the gear rotation drives the two-phase flow to generate strong eddy current, high speed field and centrifugal force field, and then pressure difference force and viscous force are generated on the gear surface, resulting in windage losses^3^. Doi^4^ showed that the oil jet location and amount of oil flow to the gears varies nearly linearly with gear pitch line velocity for high-speed, heavy-duty gears. Traditionally, the position and orientation layout of oil jet nozzles has a significant influence on the lubrication performance. However, with the increasing rotating speed of aviation gears, the impact of windage resistance effect caused by the hydrodynamic behavior on the trajectory of the oil jet stream must be paid enough attention^5,6^.

Based on experiments and analysis, researchers have put forward many findings and achievements about gear windage power loss. Anderson^7,8^ proposed an approximate method for estimating windage loss of spur gears. This method considers factors such as gear structure, working conditions, and fluid properties. However, the pitch speed of the experimental gear is limited to 40 m/s. Dawson^9^ measured the windage power loss of spur and helical gears with a diameter range of 300 ~ 1600 mm in a single-phase air flow with the rotating speed of 1500 rpm by physical experiments. Diab^10,11^ conducted an experimental study on the windage power loss of spur/helical gears. The diameter range of gears is 144 ~ 300 mm. By introducing dimensionless terms representing fluid flow and gear geometry, an empirical equation and a quasi-analytical power loss formula are obtained. Seetharaman^12^ established a simplified hydrodynamic model for spur gear windage loss to explain the suction/squeeze effect of gear meshing on windage loss. Townsend^13^ identified the variables that affect windage loss, especially gear diameter, rotational speed, housing, oil supply mode, operating conditions, and fluid viscosity. In addition, back-flow design and lubrication flow rate are also critical in controlling windage, as they directly control the flow field characteristics around rotating gears. Heingartner^14^ carried out many windage tests and proposed a formula for calculating the windage loss of helical gears. Ruzek^15,16^ used an improved special test-bench to study the air resistance power loss of disc, spur gear and helical gear under five different working conditions through a series of experiments. The results show that the total windage loss of the meshing gear pair is about equal to the sum of the windage losses when two gears are not engaged. Delgado^17^ obtained a relatively comprehensive data set of windage loss through the test facility of NASA Glenn Research Center. The effects of spur gear geometry, shroud geometry, lubrication configurations, system pressure, temperature, and gear meshing on windage loss were studied.

In reference^18^ the oil jet impingement depth on the gear surface was first introduced to investigate the lubrication of Ryder gears on the disengaging side. A positive correlation between the oil jet impingement depth and lubrication was found. Akin^19–21^ carried out many experiments on spur gear oil injection lubrication by changing gear geometric parameters and oil injection velocity. It is proved that the trajectory of small droplets will be obviously affected by fluid windage. However, the windage torque caused by the interaction between oil and gears and the influence of windage on jet trajectory are not studied in their papers. Wu^22,23^ studied the deviation phenomenon of liquid jet in crossflow and the influencing factors of oil jet trajectory. It is concluded that the crossflow Weber number is the parameter that controls the breakup regime. And the jet impingement depth will increase with increasing of oil-gas momentum ratio. Massini et al.^24,25^ designed a new rotary test rig to simulate the lubrication phenomenon of oil jet. The oil injection jet is generated by a nozzle and radially impact a single spur gear, to study the parameters that affect the behavior of injection jet. Based on the VOF model, Fondelli^26,27^ conducted a detailed numerical study on the radial impact of an oil injection jet on a single spur gear using the fluid volume (VOF) model and obtained the influence law of the oil-jet angle on windage torque and lubrication performance. Handschuh^28,29^ studied the influence of injection position and velocity on the temperature field of the spiral bevel gear body through many experiments. The results showed that the temperature rise of the pinion teeth was greatly affected. Wang^30,31^ employed a multiphase flow model to calculate the influence of spin flow on lubricating oil jet and proposed a design method of oil injection parameters. The jet deviation phenomenon on the engaging side is studied, and a scheme to reduce the jet deviation and dispersion is established. Zhu^32^ and Dai^33^ firstly established a mathematical model for calculating the exact impingement depth for spiral bevel gear and helical gear with oil jet lubrication, respectively. But they did not consider the effect of windage resistance on the calculation of the injection impingement depth.

At present, the research on the calculation method of oil jet lubrication windage impingement depth does not consider optimization design of nozzle layout. Or the studies on the optimized oil jet layout for gears do not consider the influence of windage resistance effect. Therefore, in the respect of trying to provide theoretical guidance for the nozzle layout, this paper establishes a calculation model of impingement depth for aviation gear pair considering windage effect.

## Oil jet lubrication and windage effect of aviation gears

### Motion analysis of oil jet stream

**Fig. 1:**
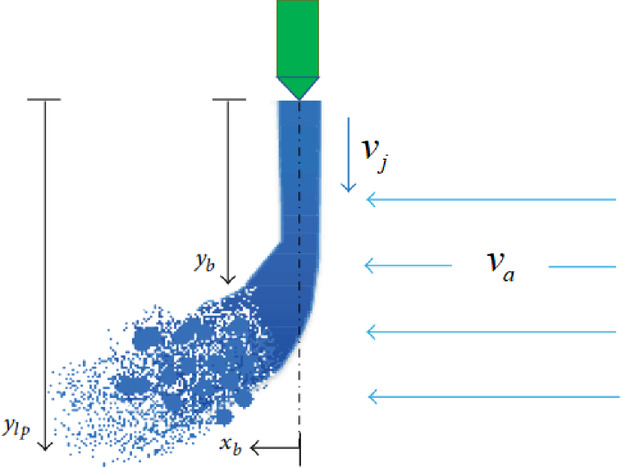
Sketch of liquid jet in the high-speed crossflow.

The lubricant must cross a rotating airflow field around the tooth top before to impact on the gear teeth. Therefore, it is necessary to consider whether the jet streamline will breakup before it impacts on the tooth flank. According to the study of Wu^22^ on the breakup of liquid jets in crossflow, the high-speed gear injection jet can be treated as an injection of liquid into a high-speed crossflow, as shown in Fig. 1.

For this phenomenon, many empirical correlations have been developed to predict the liquid column trajectory, breakup distance *x*_*b*_, and height *y*_*b*_, for various flow conditions. The main parameter governing the phenomenon is the liquid to gas momentum ratio *q* while the crossflow Weber number We_*a*_ is the parameter that controls the breakup regime^23^:

1 $$\left\{ \begin{gathered} q=\frac{{{\rho _j}{v_j}^{2}}}{{{\rho _a}{v_a}^{2}}} \hfill \\ {\text{W}}{e_a}=\frac{{{\rho _a}{d_j}{v_a}^{2}}}{\sigma } \hfill \\ \frac{{{y_b}}}{{{d_j}}}=k{q^n} \hfill \\ \end{gathered} \right.$$

where, *ρ*_*j*_ and *ρ*_*a*_ are the density of oil and air, respectively; *v*_*j*_ and *v*_*a*_ are oil jet velocity and boundary layer airflow velocity, respectively; *d*_*j*_ is nozzle diameter; *σ* is the surface tension coefficient of air; *k* and *n* are constant values.

Since the distance between the jet inlet and the gear surface of the research object in this paper is very small, and the air density is small under sub-atmospheric pressure. So, the aerodynamic force acting on the lubricant flow is significantly reduced. Therefore, it is expected that no obvious oil jet breakup will occur before the jet impacts on the gear tooth. This is consistent with the experimental results of Akin^20^.

### Influence of gear windage effect on oil jet stream

**Fig. 2:**
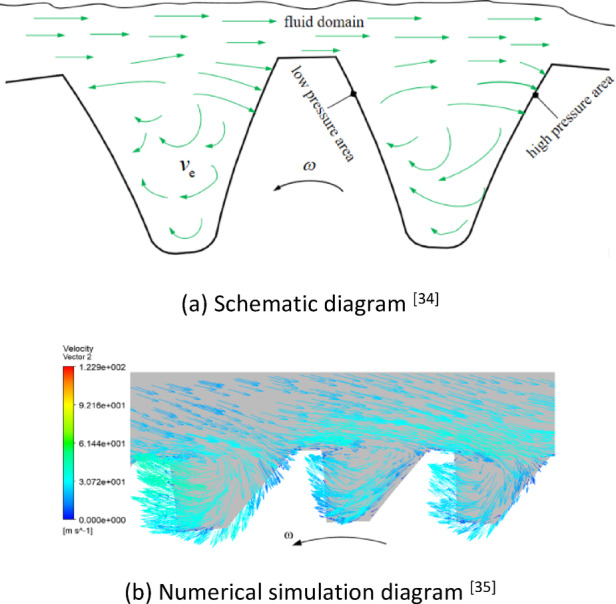
The eddy windage effect within the tooth space. (**a**) Schematic diagram^34^. (**b**) Numerical simulation diagram^35^.

The trajectory of the oil jet ligaments and droplets sprayed from the oil-jet nozzle through the tooth surface and tooth space is very complex. This is mainly due to the high-speed rotation of the aviation gear pushing and dragging the mixture of the surrounding lubricating oil and air, resulting in intense turbulent motion, thereby forming strong eddy currents between the gear teeth, as shown in Fig. 2.

The fluid around the gear is separated in a certain area of the tooth top. Some fluid flows through the tooth top without changing direction, and the other part is sucked into the space between the teeth from the position close to the tooth top, forming eddy currents in the tooth space. The tooth surface collided by fluid is manifested as the pressure surface showing local high pressure, while the leeward surface is manifested as the suction surface showing local low pressure, resulting in pressure difference between the two tooth surfaces and the formation of differential pressure moment, which is opposite to the rotation direction of the gear. This is the mechanism of windage resistance effect.

It is known that the jet-line of oil jet lubrication needs to pass through a rotating high-speed windage flow field before it can impact on the teeth and finally enter the tooth space. As studied by Marchesse^36^, Hill^37^, and Albertson^38^.

**Fig. 3:**
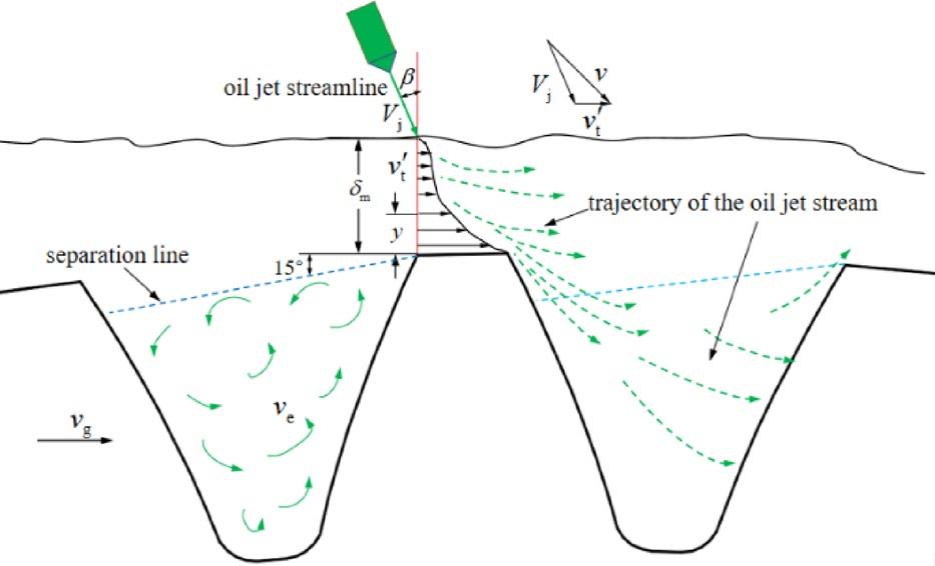
Sketch of oil jet ligament moving past eddy windage.

The windage boundary layer shown in Fig. 3 involves the entire periphery of the gear wheel as represented by the outermost wavy line which represents the maximum boundary thickness *δ*_m_. Where the velocity *v*_t_’ is equal to 1% of the gear velocity. The velocity *v*_t_’ in any position on the velocity profile can be calculated from Prandtl’s one-seventh power law.

2 $${v_t}^{\prime }={v_g}\left[ {1 - {{\left( {\frac{y}{{{\delta _m}}}} \right)}^{{\raise0.5ex\hbox{$\scriptstyle 1$}\kern-0.1em/\kern-0.15em\lower0.25ex\hbox{$\scriptstyle 7$}}}}} \right]$$

where, *v*_g_ is the gear pitch velocity, and *y* is the distance between the fluid and the tooth top surface.

**Fig. 4:**
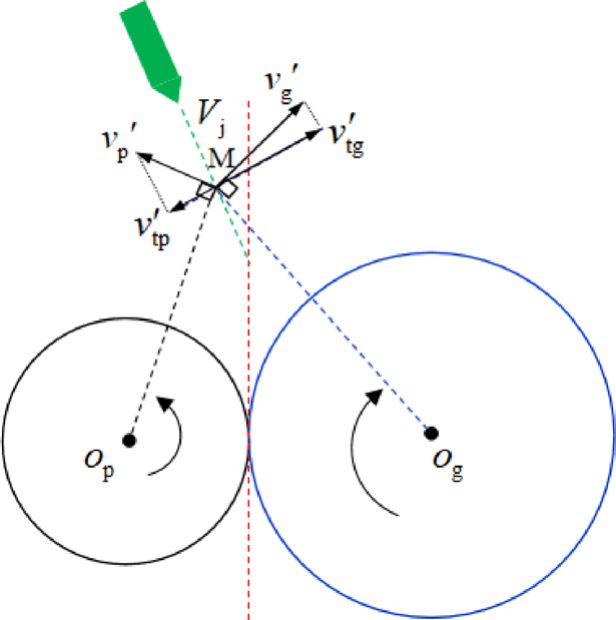
Oil jet deviation of gear pair in the out-of-mesh condition.

The motion trajectory of the oil jet line is deflected when it flows through the windage boundary layer, which indicates that the windage effect in a certain area outside the gear produces a deflection effect on the direction of the oil injection jet. The cause of oil jet deviation of gear pair is shown in Fig. 4. By analyzing the motion state of the fluid at any point M on the trajectory of the jet line, the fluid at M has two tangential velocities, *v*_p_’ and *v*_g_’, due to the respective rotating flow fields of the pinion and gear. The magnitude of *v*_g_’ is greater than *v*_p_’ known from Eq. (2), so that the velocity component perpendicular to the oil jet direction, *v*_tg_’ is greater than *v*_tp_’, which leads to the tendency of the fluid at point M to sling oil to the driven gear when the lubricant jet flow is in the out-of-mesh condition.

## Calculation model of oil jet windage impingement depth

### Layout parameters and constraints of oil jet streamline

**Fig. 5:**
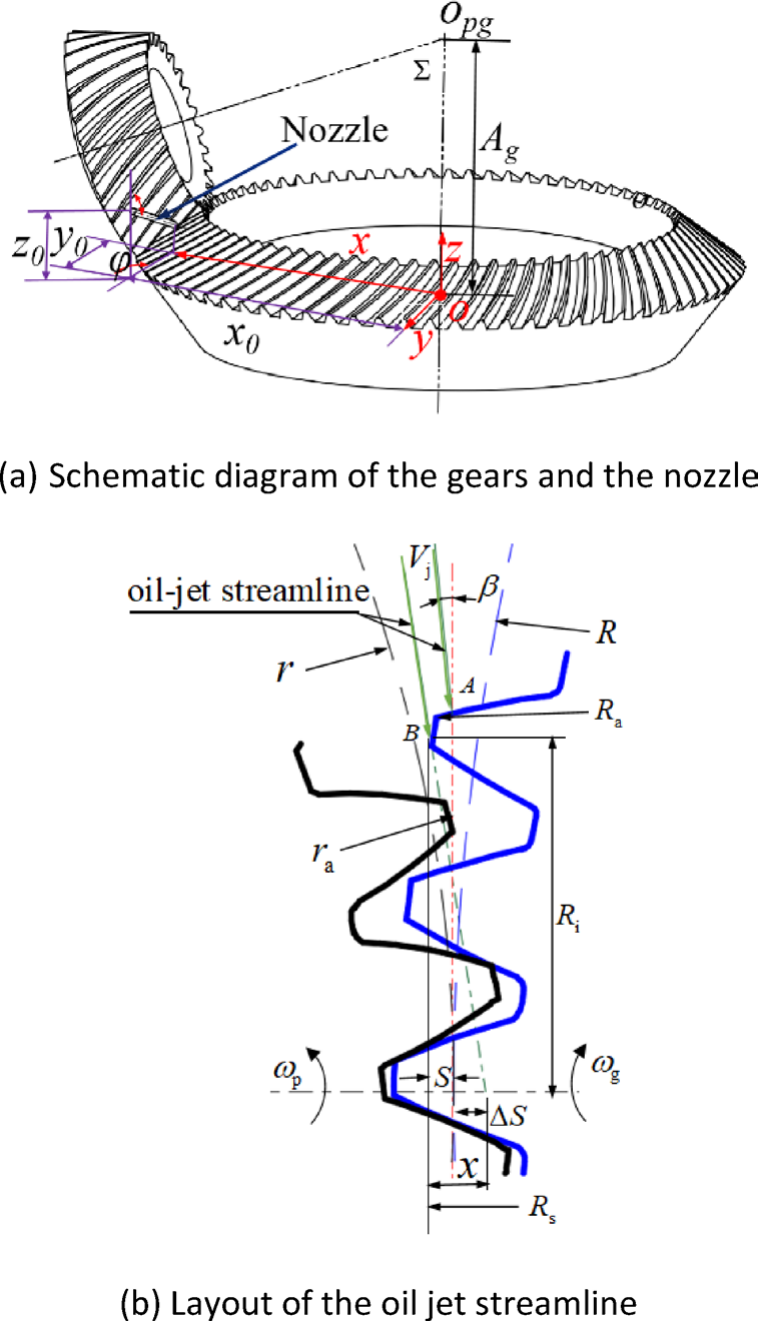
Layout parameters. (**a**) Schematic diagram of the gears and the nozzle. (**b**) Layout of the oil jet streamline.

The layout parameters of the jet streamline for the gear pair mainly include initial offset distance of jet stream trajectory *S*, inclination angle *β*. The geometric definition of *S* and *β* are described below and shown in Fig. 5. The initial offset *S* is defined as the straight-line distance between the impingement point and the common tangent of the pitch circle of the gear pair. The inclination angle *β* represents the acute angle between the oil jet line and the common tangent of the gear pitch circle. *β* is considered positive when slanted to the pinion and negative when slanted to the gear.

It can be seen from Fig. 5 that when the impact point between the injection line and the gear is the intersection of the tooth profile and the common tangent of the pitch circle, *S* = 0; When the impact point between the oil jet streamline and the gear is the outermost edge point of the driven gear top contour, *S* = *S*_*max*_, and:

3 $$\begin{gathered} {R_{\text{i}}}={\left( {R_{a}^{2} - R_{s}^{2}} \right)^{1/2}} \hfill \\ {R_{\text{s}}}=R+S \hfill \\ \end{gathered}$$

where *h*_*a*_ represents the tooth addendum, and *u* is the gear reduction ratio.

When the parameter *S* is positive, it cannot obviously reflect the offset direction of the jet streamline. So, the parameter Δ*S* is introduced to represent the oil jet streamline offset. Δ*S* is defined as the distance from the intersection point of the jet line and the gear pair center line to the common tangent of the gear pair pitch circle, as shown in Fig. 5, and

4 $$\Delta S=x - S$$

where, *x* is the arbitrary offset distance from S position to where the jet stream crosses the common line of centers.

To facilitate calculation, dimensionless processing of *S* can be obtained as follows:

5 $${S_i}=\frac{S}{{{S_{\hbox{max} }}}}$$

The mathematical definition of the arbitrary inclination angle *β* is:

6 $$\beta =\arctan \frac{x}{{{R_i}}}$$

where, *R*_*i*_ is the vertical distance between the impingement point and the common line of centers, and

7 $$\begin{gathered} {R_i}={\left( {R_{a}^{2} - R_{s}^{2}} \right)^{1/2}} \hfill \\ {R_s}=R+S \hfill \\ \end{gathered}$$

where, *R*_*a*_ and *R* are the outside radius and pitch radius of the gear, respectively. *R*_*s*_ is the offset radius between the jet streamline and the center point of the gear.

When the jet streamline and the center line intersect at the top circle of the pinion and the gear respectively, *β* has the maximum and the minimum values, and

8 $$\left\{ \begin{gathered} {\beta _{\hbox{max} }}=arc\tan \frac{{S+{h_{pa}}}}{{{R_i}}} \hfill \\ {\beta _{\hbox{min} }}=arc\tan \frac{{S - {h_{ga}}}}{{{R_i}}} \hfill \\ \end{gathered} \right.$$

where, *h*_*p.a.*_ and *h*_*ga*_ are the addendum of the pinion and gear, respectively.

Similarly, *β*_*i*_ is obtained by dimensionless treatment of *β*, and − 1 ≤ *β*_*i*_ ≤ 1. When *β*_*i*_ is positive:

9a $${\beta _i}=\frac{{\beta - \beta ^{\prime}}}{{{\beta _{\hbox{max} }} - \beta ^{\prime}}}$$

when *β*_*i*_ is negative:

9b $${\beta _i}=\frac{{\beta - \beta ^{\prime}}}{{\beta ^{\prime} - {\beta _{\hbox{min} }}}}$$

where, *β’* is the value of *β* when the radiation line and the center line intersect at the node of two gear indexing circles, and its value is:

9c $$\beta ^{\prime}=arc\tan \frac{S}{{{R_i}}}$$

Since the ratio of oil jet velocity to linear velocity of the gear pair is also an important factor affecting the lubrication effect of gear pair. So, the oil jet velocity *V*_*j*_ is dimensionless processed into *V*_*i*_:

10 $${V_i}=\frac{{{V_j}}}{{{v_p}}}$$

where, *V*_*j*_ is the oil jet velocity and *v*_*p*_ is pitch velocity of the pinion.

### The definition of the windage impingement depth

**Fig. 6:**
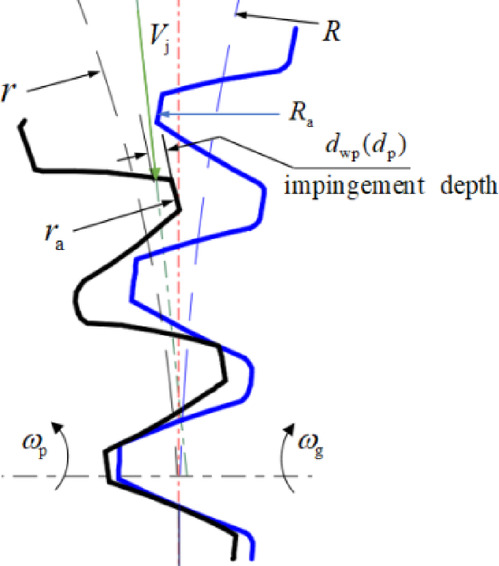
The schematic diagram of the impingement depth.

“Windage impingement depth” is defined as the straight-line distance from the impingement point of the oil jet line on the gear profile to the tooth top, indicating the depth of the fluid reaching the tooth space under the action of the radial momentum, viscous resistance, and centrifugal force field of the fluid after the lubricating oil impacts the gear teeth. The schematic diagram of the definition of the impingement depth *d*_p_ or *d*_wp_ is shown in Fig. 6, when the oil jet streamline impacts the pinion. The initial impact point between oil jet flow and tooth profile and the impingement depth after entering the tooth space are important factors to determine the effect of gear cooling and lubrication.

In the process of oil jet lubrication, when the jet streamline passes through the boundary layer of windage, its motion trajectory will be deflected. When the jet streamline impinges on the tooth profile, the windage vortex in the tooth space will also affect the trajectory and distribution of lubricating oil in the tooth space. Therefore, the windage effect of the rotating flow field of the gear pair cannot be ignored.

### Mathematical model of windage impingement depth for the pinion

**Fig. 7:**
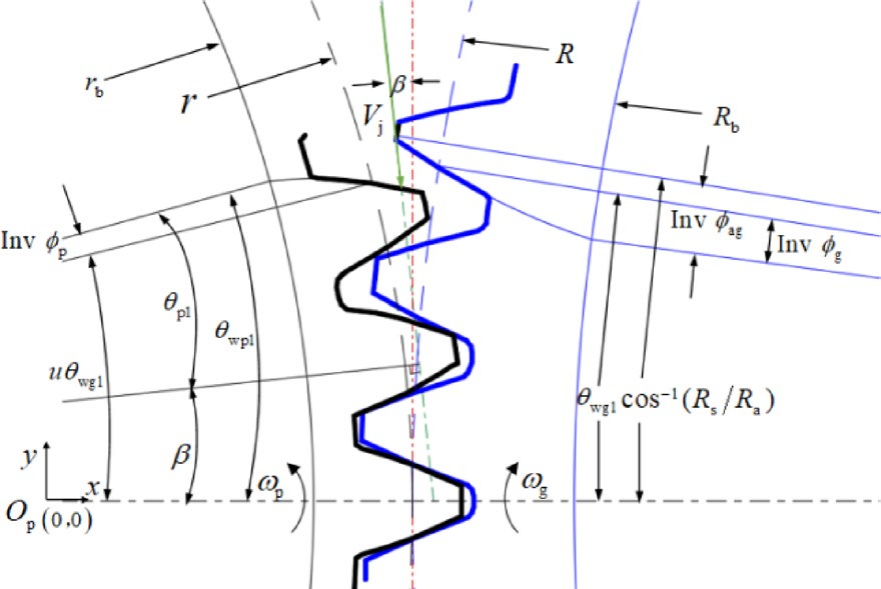
Impingement on pinion at *t* = 0.

Windage resistance of the rotating flow field around gears affects the motion trajectory of the oil jet streamline, which in turn influences the distribution of the lubricating oil flow on the tooth surface, and ultimately determines the lubrication and cooling effect of gears. This section mainly considers the motion of the oil droplets on the tooth space with windage effect. When the jet reaches the trailing edge of the gear tooth as shown in Fig. 7 and the coordinate origin is set at the center of the pinion, it is the position of the pinion impingement cycle *t* = 0.

In Fig. 7, at the initial time *t* = 0, *θ*_*wp*1_and *θ*_*wg*1_ are respectively the geometrical position of the pinion and gear, and:

11 $$\begin{gathered} {\theta _{wp1}}=u{\theta _{wg1}}+inv{\phi _p} \hfill \\ {\theta _{wg1}}=\arccos \frac{{{R_s}}}{{{R_a}}} - inv{\phi _{ag}}+inv{\phi _g} \hfill \\ \end{gathered}$$

Where, *ϕ*_*p*_ and *ϕ*_*g*_ are the pressure angles at the pitch radius of the pinion and gear, respectively. *ϕ*_*ag*_ is the pressure angles at the outside diameter of the gear.

A droplet on the jet streamline was taken as the research object. It is assumed that $\sum {F_{\text{z}}}$ can be neglected in spur gears as a second order effect and the actual involute gear tooth can be treated as a rack-tooth profile. The dynamic equation of the droplet is:

12 $$\left\{ \begin{gathered} \sum {F_{\text{x}}}= - {C_{\text{D}}}\frac{{\pi d_{{\text{o}}}^{2}}}{4}{\rho _{\text{m}}}\frac{{v_{{\text{r}}}^{2}}}{2} - {\rho _o}\frac{{\pi d_{{\text{o}}}^{3}}}{6}{a_{\text{r}}}=0 \hfill \\ \sum {F_{\text{y}}}={C_{\text{D}}}\frac{{\pi d_{{\text{o}}}^{2}}}{4}{\rho _{\text{m}}}\frac{{{{\left( {{v_g} - {v_t}} \right)}^2}}}{2}+{\rho _{\text{o}}}\frac{{\pi d_{{\text{o}}}^{3}}}{6}{a_{\text{t}}}=0 \hfill \\ \end{gathered} \right.$$

where, *C*_D_ is fluid drag coefficient, and *d*_o_ is droplet diameter, and *ρ*_m_ and *ρ*_o_ are density of oil-gas two-phase flow and droplet, respectively; *v*_r_, *v*_t_ and *a*_r_, *a*_t_ are the velocity and acceleration components of the droplet in the *x* and *y* directions, respectively. The calculation formula for the mixing density of oil and gas two-phase flow is:

13 $${\rho _{\text{m}}}{\text{=}}\frac{{{\rho _g}{\rho _{\text{o}}}}}{{{\rho _{\text{o}}}(1 - {f_{\text{o}}})+{\rho _g}{f_{\text{o}}}}}$$

where, *ρ*_g_ is the density of air and *f*_o_ is the volume fraction of lubricating oil.

The initial velocity and position coordinates of the droplet are:

14 $$\begin{gathered} \left\{ \begin{gathered} {v_{{\text{r0}}}}={V_{\text{j}}}\sin \beta \hfill \\ {v_{{\text{t0}}}}= - {V_{\text{j}}}\cos \beta \hfill \\ \end{gathered} \right. \hfill \\ \left\{ \begin{gathered} {x_{\text{0}}}=r - S \hfill \\ {y_{\text{0}}}={\left( {R_{{\text{a}}}^{2} - {{\left( {R+S} \right)}^2}} \right)^{1/2}} \hfill \\ \end{gathered} \right. \hfill \\ \end{gathered}$$

where, *v*_r0_, *v*_t0_ and *x*_0_, *y*_0_ are the velocity components and position coordinates of the droplet in the *x* and *y* directions at the initial time, respectively. *r* is pitch radius of the pinion. *V*_j_ is the jet velocity.

So, the acceleration components of the droplet in the *x* and *y* directions can be obtained by simplifying Eq. (12):

15 $$\left\{ \begin{gathered} {a_{\text{r}}}{\text{=}}{\raise0.7ex\hbox{${\partial {v_{\text{r}}}}$} \!\mathord{\left/ {\vphantom {{\partial {v_{\text{r}}}} {\partial t}}}\right.\kern-0pt}\!\lower0.7ex\hbox{${\partial t}$}}= - \left( {\frac{{3{C_D}{\rho _m}}}{{4{d_{\text{o}}}{\rho _{\text{o}}}}}} \right)v_{{\text{r}}}^{2}= - \gamma v_{{\text{r}}}^{2} \hfill \\ {a_{\text{t}}}{\text{=}}{\raise0.7ex\hbox{${\partial \left( {{{{v_{\text{t}}}} \mathord{\left/ {\vphantom {{{v_{\text{t}}}} {{v_{\text{g}}}}}} \right. \kern-0pt} {{v_{\text{g}}}}}} \right)}$} \!\mathord{\left/ {\vphantom {{\partial \left( {{{{v_{\text{t}}}} \mathord{\left/ {\vphantom {{{v_{\text{t}}}} {{v_{\text{g}}}}}} \right. \kern-0pt} {{v_{\text{g}}}}}} \right)} {\partial t}}}\right.\kern-0pt}\!\lower0.7ex\hbox{${\partial t}$}}={\raise0.7ex\hbox{${\partial {v_{\text{i}}}}$} \!\mathord{\left/ {\vphantom {{\partial {v_{\text{i}}}} {\partial t}}}\right.\kern-0pt}\!\lower0.7ex\hbox{${\partial t}$}}= - \left( {\frac{{3{C_D}{\rho _m}}}{{4{d_{\text{o}}}{\rho _{\text{o}}}}}} \right){v_{\text{g}}}\left( {1 - v_{{\text{i}}}^{2}} \right) \hfill \\ {\text{ }}= - \gamma {v_{\text{g}}}\left( {1 - v_{{\text{i}}}^{2}} \right) \hfill \\ \end{gathered} \right.$$

where, $$\gamma {\text{=}}\frac{{3{C_D}{\rho _m}}}{{4{d_{\text{o}}}{\rho _{\text{o}}}}}$$, $${{{v_{\text{i}}}{\text{=}}{v_{\text{t}}}} \mathord{\left/ {\vphantom {{{v_{\text{i}}}{\text{=}}{v_{\text{t}}}} {{v_{\text{g}}}}}} \right. \kern-0pt} {{v_{\text{g}}}}}$$.

Integrate the Eq. (15), and combine with the initial condition Eq. (14) to get:

16 $$\left\{ \begin{gathered} {v_{\text{r}}}{\text{=}}{\raise0.7ex\hbox{${{d_{\text{x}}}}$} \!\mathord{\left/ {\vphantom {{{d_{\text{x}}}} {{d_{\text{t}}}}}}\right.\kern-0pt}\!\lower0.7ex\hbox{${{d_{\text{t}}}}$}}=\frac{{{V_{\text{j}}}\sin \beta }}{{\gamma {V_{\text{j}}}\sin \beta t+1}} \hfill \\ {v_{\text{t}}}{\text{=}}{\raise0.7ex\hbox{${{d_{\text{y}}}}$} \!\mathord{\left/ {\vphantom {{{d_{\text{y}}}} {{d_{\text{t}}}}}}\right.\kern-0pt}\!\lower0.7ex\hbox{${{d_{\text{t}}}}$}}{\text{=}}{v_{\text{g}}} - \frac{1}{{\gamma t+{1 \mathord{\left/ {\vphantom {1 {\left( {{v_{\text{g}}}+{V_{\text{j}}}\cos \beta } \right)}}} \right. \kern-0pt} {\left( {{v_{\text{g}}}+{V_{\text{j}}}\cos \beta } \right)}}}} \hfill \\ \end{gathered} \right.$$

Integrate the Eq. (16), and combine with the initial condition Eq. (14) to get the position coordinates of the droplet at any time:

17 $$\left\{ \begin{gathered} x(t)=\frac{1}{\gamma }\ln \left( {\gamma {V_{\text{j}}}\sin \beta t+1} \right)+r - S \hfill \\ y(t){\text{=}}{v_{\text{g}}}t - \frac{1}{\gamma }\ln \left( {\gamma t+{1 \mathord{\left/ {\vphantom {1 {\left( {{v_{\text{g}}}+{V_{\text{j}}}\cos \beta } \right)}}} \right. \kern-0pt} {\left( {{v_{\text{g}}}+{V_{\text{j}}}\cos \beta } \right)}}} \right) \hfill \\ {\text{ +}}{{\text{R}}_{\text{i}}}+\frac{1}{\gamma }\ln \left( {{1 \mathord{\left/ {\vphantom {1 {\left( {{v_{\text{g}}}+{V_{\text{j}}}\cos \beta } \right)}}} \right. \kern-0pt} {\left( {{v_{\text{g}}}+{V_{\text{j}}}\cos \beta } \right)}}} \right) \hfill \\ \end{gathered} \right.$$

**Fig. 8:**
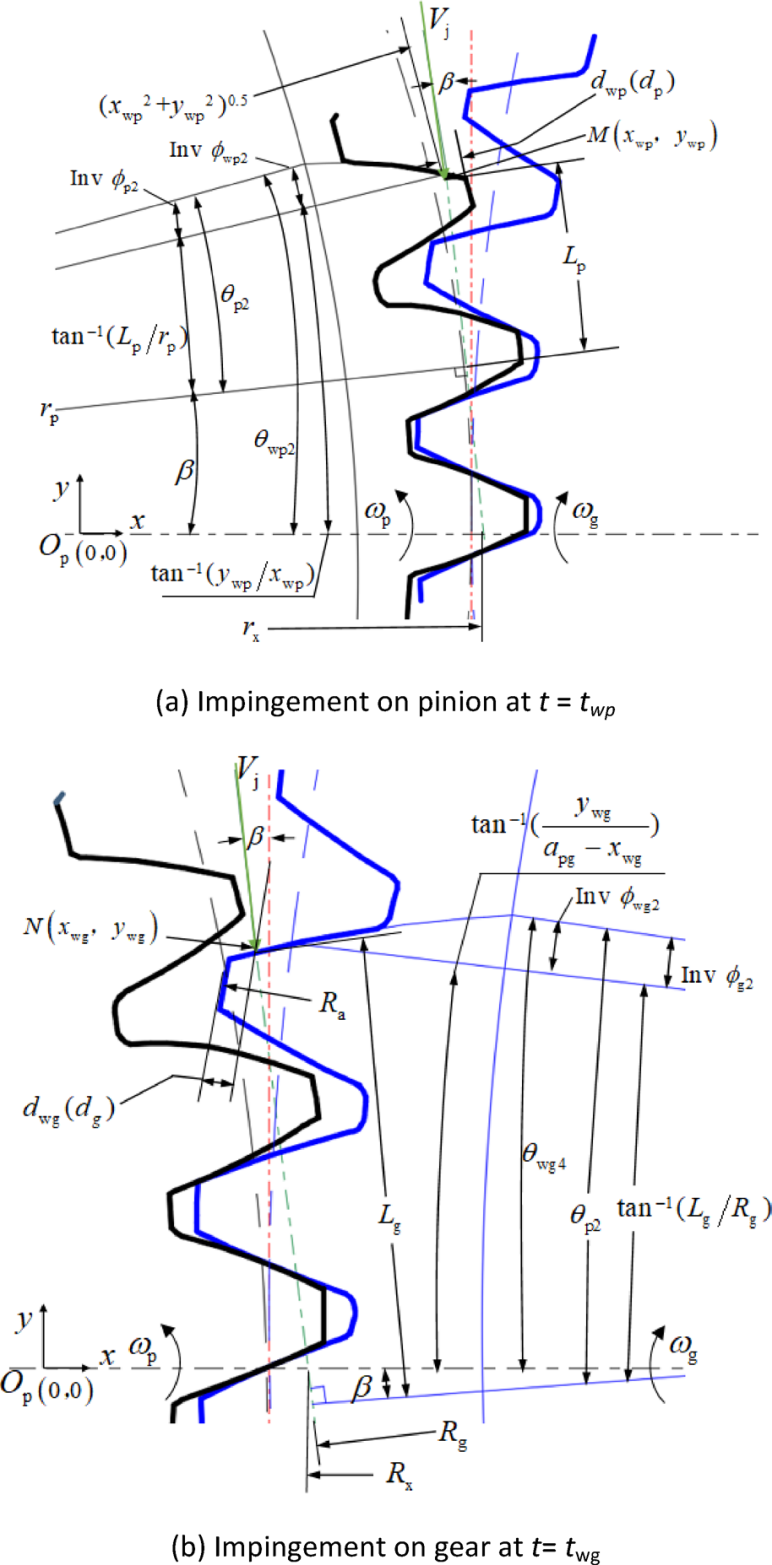
Impingement on gear pair. (**a**) Impingement on pinion at *t* = *t*_*wp*_. (**b**) Impingement on gear at *t* = *t*_wg_

It is assumed that at time *t*_wp_, the droplet impinges with the tooth profile of the pinion at point *M* (*x*_wp_, *y*_wp_), as shown in Fig. 8a.

At this time, it can be seen from the geometric relationship in Fig. 8 that the angular position of the pinion is:

18 $${\theta _{{\text{wp2}}}}{\text{=}}arctan\frac{{{y_{{\text{wp}}}}}}{{{x_{{\text{wp}}}}}}+inv{\phi _{{\text{wp2}}}}$$

where, *ϕ*_wp2_ is the pressure angle at impingement point *M*, and

19 $${\phi _{{\text{wp2}}}}=arccos\frac{{{r_{\text{b}}}}}{{{{\left( {x_{{{\text{wp}}}}^{2}+y_{{{\text{wp}}}}^{2}} \right)}^{0.5}}}}$$

where, *r*_b_ is the base radius of pinion.

From time *t* = 0 to *t* = *t*_wp_, the pinion rotates from geometrical position *θ*_wp1_ to *θ*_wp2_, so there is a relationship:

20 $$\frac{{{\theta _{{\text{wp}}2}} - {\theta _{{\text{wp}}1}}}}{{{\omega _{\text{p}}}}}={t_{{\text{wp}}}}$$

where, ω_p_ is the angular velocity of the pinion.

The implicit transcendental equation for *t*_wp_ can be obtained by combining Eqs. (17), (18) and (20). When the gear pair related parameters and the oil jet streamline positioning parameters *S* and *β* are known, the approximate solution ${\tilde {t}_{{\text{wp}}}}$ of the Eq. (20) can be implicitly obtained by iteration. The position coordinates of the impingement point *x*_wp_, *y*_wp_ are provided in Eq. (17) by the approximate solution${\tilde {t}_{{\text{wp}}}}$. Then the depth of impingement on the pinion is

21 $${d_{{\text{wp}}}}={r_{\text{a}}} - {\left( {x_{{{\text{wp}}}}^{2}+y_{{{\text{wp}}}}^{2}} \right)^{{\raise0.5ex\hbox{$\scriptstyle 1$}\kern-0.1em/\kern-0.15em\lower0.25ex\hbox{$\scriptstyle 2$}}}}$$

where, *r*_a_ is the outside radius of the pinion.

Similarly, as shown in Fig. 8b, the calculation formula of the impingement depth for the gear can be obtained as:

22 $${d_{{\text{wg}}}}={R_{\text{a}}} - {\left( {{{\left( {{a_{{\text{pg}}}} - {x_{{\text{wg}}}}} \right)}^2}+y_{{{\text{wg}}}}^{2}} \right)^{{\raise0.5ex\hbox{$\scriptstyle 1$}\kern-0.1em/\kern-0.15em\lower0.25ex\hbox{$\scriptstyle 2$}}}}$$

In the aviation gear transmission system, the larger the range of the gear meshing surface being sprayed by lubricating oil along the tooth height direction, the more conducive it is to form oil film and take away heat^18^. So, the impingement depth calculation model of gear pair is obtained.

## CFD numerical simulation model

### Fluid control equation

The oil-jet lubrication process of gear pair is an oil-gas two-phase flow problem, which requires multiphase numerical calculation analysis. So, the homogeneous model of Euler-Euler VOF two-phase flow modeling is necessary to capture oil flow behavior^27^. In the VOF model, it is assumed that each phase fluid in each computational unit has the same velocity and pressure, and the mixture composed of each phase fluid is regarded as homogeneous flow. The same momentum equations are solved for the insoluble fluid and the volume fraction of each fluid is calculated in the whole computational domain. Model control equation is:

(1) Volume conservation equation 

23 $$\sum\limits_{{q=1}}^{N} {{\alpha _{\text{q}}}} =1$$

where, the lowercase letter *q* is the fluid phase, *q* are oil and air here. *α*_*q*_ is the volume fraction of *q* phase. *N* is the number of fluid phases, here *N* = 2.

(2) Quality conservation equation 

24 $$\frac{{\partial \rho }}{{\partial t}}+\frac{{\partial \left( {\rho u} \right)}}{{\partial x}}+\frac{{\partial \left( {\rho v} \right)}}{{\partial y}}+\frac{{\partial \left( {\rho w} \right)}}{{\partial z}}={\Gamma _{q1q2}} - {\Gamma _{q2q1}}$$

where, $\rho {\text{=}}\sum\limits_{{q=1}}^{N} {{\alpha _q}} {\rho _q}$ is the mixed fluid density, ${\rho _q}$ is the density of *q* phase. *t* is time. *u*,* v*,* w* are the components of the fluid velocity along the *x*, *y* and *z* axes respectively. ${\Gamma _{q1q2}}$ is the positive interphase mass flow rate per unit volume from phase *q*1 to *q*2.

(3) Momentum conservation equation 

25 $$\frac{{\partial (\rho U)}}{{\partial t}}+\nabla \cdot (\rho UU)= - \nabla P+\nabla \cdot \tau +\rho g+F$$

where, $U{\text{=}}ui+vj+wk$ is flow velocity. *P* is mixed fluid pressure. *F* represents external body forces. $\nabla$ is divergence. *τ* is the viscous stress tensor, and the calculation formula is

26 $$\tau =\mu \left[ {\nabla U+{{\left( {\nabla U} \right)}^T} - \frac{2}{3}\delta {\text{ }}\nabla \cdot U} \right]$$

where *µ* is the hydrodynamic viscosity.

### Fluid field model

The basic parameters of aviation spur gear pair are shown in Table 1.

**Table 1:** The basic parameters of aviation gear pair.

Parameters	Pinion	Gear
Number of teeth *z*	56	92
Speed *n*/(rpm)	1000 ~ 11,000	-
Module *m*/(mm)	2.54	
Pressure angle *α*/(°)	22.5	
Tooth width *B*/(mm)	6.5	6.1

**Fig. 9:**
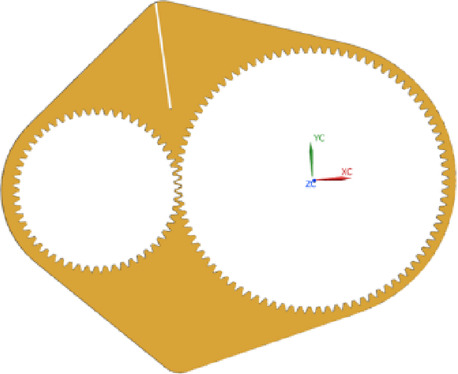
Fluid field model of gearbox.

The oil-jet nozzle is installed on the central plane of the tooth width. The gearbox model with the oil-jet nozzle is created in UG. The distance between the end face of the gear and the wall of the gearbox is 2 mm, the diameter of the nozzle is 2 mm, and the diameter of the oil outlet is 22.5 mm. To reduce the simulation difficulty, reduce unnecessary computing resources and improve the simulation efficiency, the gearbox model is properly simplified. The gear shaft, bearing, pipe, small fillet and various chamfer is ignored. Extract the internal flow field of gearbox and do not consider the tiny structure of gearbox. Boolean operation in UG is used to obtain the flow field model. The cross-section model of the fluid domain through the tooth width center plane is shown in Fig. 9.

### Meshing and solution settings

The gearbox geometry model was imported into the pre-processing software ANSYS-ICEM CFD, and the fluid domain was discretized into unstructured tetrahedral meshes based on the finite volume method, to obtain the fluid dynamics analysis model. In order to ensure the accuracy of CFD calculation, reduce the calculation time and save the calculation resources, it is necessary to locally densify and refine the mesh on each surface of the gear and near the oil injection nozzle to ensure that there are at least 2 ~ 3 layers of mesh elements at the minimum gap of the gear meshing area. At the same time, in order to obtain high-quality mesh, it is necessary to smooth the mesh. The maximum mesh size of the fluid domain is 3 mm, the boundary layer size is 0.4 mm at tooth surface, end face and nozzle, and the thickness of boundary layer is 5 layers. Finally, the number of mesh elements in the entire fluid domain of the calculation model is about 10.24 million and about 1.57 million nodes.

## The multi-objective optimization model of oil jet streamline layout parameters

### Establishing multi-objective optimization model

To ensure that there is enough lubricating oil in the meshing area to cool and lubricate the teeth, the impingement depth value of the two gears can be maximized and the difference between them can be minimized through the optimal oil jet streamline layout, that is, both gears can reach the relatively optimal impingement depth value at the same time. Because there is a specific speed threshold where the effect of windage resistance becomes significant and cannot be ignored. The Table 2 is the test result of gearbox lubrication performance in a literature.

**Table 2:** The oil film thickness test results of a gearbox at different speeds.

Speed (rpm)	Oil film thickness (µ)	Windage effect
500	0.8	negligible
3000	1.5	lesser
8000	1.8	significant
12,000	1.2	serious

As can be seen from the table, when the gear speed is about 8000 rpm, the oil film thickness reaches the maximum and then begins to decrease, and the influence of windage becomes significant and cannot be ignored. Also, from reference^39^ we can see that when the gear speed is 7000 ~ 8000 rpm, the windage loss increases sharply, and the windage effect has a great influence on the lubrication performance, which cannot be ignored. So, the multi-objective optimization problem with multiple constraints is solved from the perspective of multiple factors, that is, taking the maximum impingement depth of gear pair and the minimum difference between them as the optimization objective and the layout parameters of oil jet streamline as the constraints, and the impingement depth optimization design model with windage effect is established as follows:

Optimization variables $$x=[S,\beta ]$$

Optimization objectives

27 $$\left\{ \begin{gathered} {\text{Min }}{f_{\text{1}}}({\varvec{x}})= - {F_1}({\varvec{x}}) \hfill \\ {\text{Min }}{f_{\text{2}}}({\varvec{x}})= - {F_{\text{2}}}({\varvec{x}}) \hfill \\ {\text{Min }}{f_3}({\varvec{x}})=\left| {{f_{\text{1}}}{\text{(}}x{\text{)}} - {f_{\text{2}}}{\text{(}}x{\text{)}}} \right| \hfill \\ \end{gathered} \right.$$

where, *x* is the jet layout parameters; *F*_1_(*x*) and *F*_2_(*x*) are the depth of impingement on the pinion and gear calculated by Eqs. (21) and (22).

### The multi-objective optimization method

**Fig. 10:**
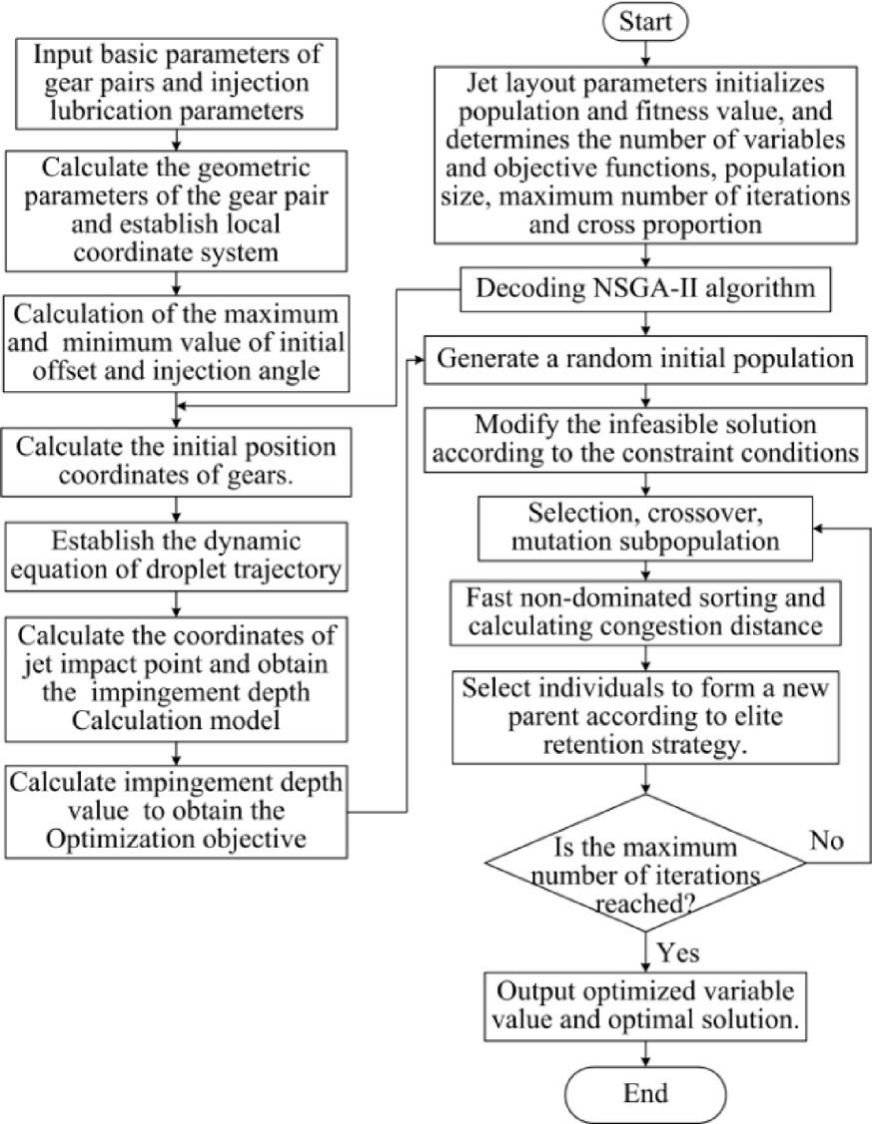
Multi-objective optimization flow chart of jet layout parameters.

Based on the dynamic theory of fluid motion, the calculation model of impingement depth is established in this paper. This paper Combines windage effect and lubrication characteristics of aviation gear pair and uses multi-objective NSGA-II algorithm to optimize the jet layout parameters. The flow chart of iterative optimization process is shown in Fig. 10.

**Fig. 11:**
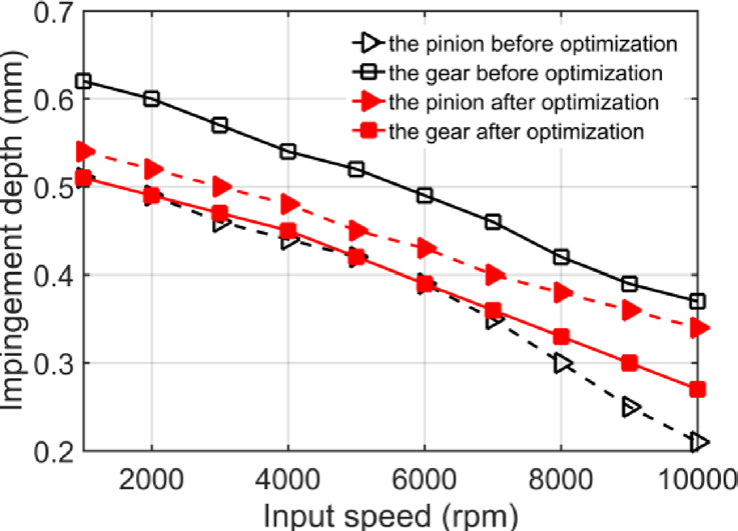
Variation curve of jet depth with input speed before and after optimization.

Taking the aviation gear pair of Table 1 as an example, the windage impingement depth under different conditions is calculated. A lubricant oil of Mobil Jet Oil II is used here. The oil injection velocity is 40 m/s, and the injection temperature is 20℃. At this point the viscosity of lubricating oil is 0.0604 *Pa. s* and the density is 998 *kg*/*m*^3^. The droplet diameter is 0.05 mm, and the gear rotating speed is 9000 rpm. According to the NSGA-II algorithm flow in Fig. 10, the oil jet layout parameters are optimized. The population size is set to 500, the number of iterations is 500, and the cross ratio is 0.8. The optimized jet layout parameters are shown in Table 3. The curve of windage impingement depth before and after optimization with input speed is shown in Fig. 11.

**Table 3:** The optimized jet layout parameters.

Optimization parameters	*β* (°)	*S* (mm)	Δ*S* (mm)
	4.6	0.53	-0.48

The jet impingement depth values of the two gears are quite different before optimization, and the pinion is much smaller than the gear. Due to the windage effect, when the gear speed is too high, there is a situation that the lubricating oil cannot be injected onto the pinion teeth. This is mainly because that the higher rotating speed of the gear, the more intense the movement of the windage flow field around it, and the stronger the windage drag, resulting in an increase of its blocking effect on lubricating oil. What is more, when the injection velocity is relatively small, there will be large deviation or even complete contra-flow of lubricating oil flow, which will eventually lead to the failure of lubricating oil to spray onto the gear teeth, resulting in the reduction of gear efficiency and service life. Literature research show that the deeper the impingement depth of lubricating oil on the gear surface, the more lubricating oil can fully penetrate into the microstructure of the gear surface and form a uniform oil film. Oil film can effectively absorb and disperse the heat generated by friction and reduce the surface temperature of gears^34^. However, with the increase of gear speed, the impingement depth first increases and then decreases due to the windage effect, so that the gear surface temperature increases significantly at high speed. Therefore, optimizing the jet layout parameters to make the jet flow of the two gears equal, not only provides a theoretical reference for the design of gear lubrication system, but also improves the gear transmission efficiency.

## Model validation and result analysis

### Effect of gear geometry parameters

**Fig. 12:**
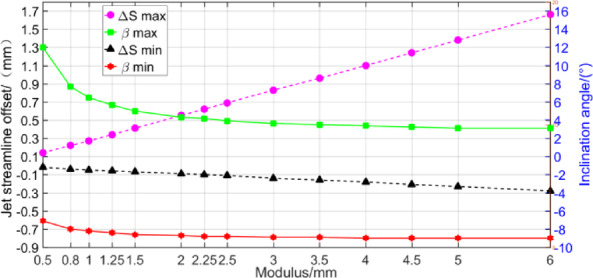
Variation of inclination angle and jet streamline offset versus modulus.

**Fig. 13:**
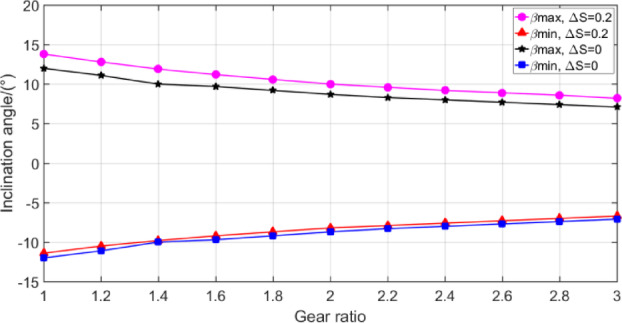
The range of *β* with different gear ratio.

**Fig. 14:**
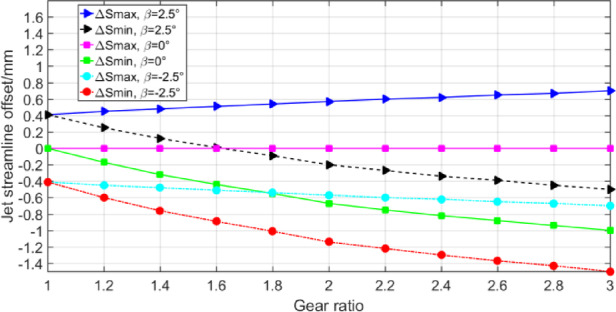
The range of *ΔS* at different gear ratio.

According to the calculation model in Sect. 3, among the geometric parameters of gears, the modulus and gear transmission ratio have the greatest influence on the oil injection impingement depth, which mainly affect the value range of oil jet inclination angle *β* and oil jet streamline offset Δ*S*. The calculation results are illustrated in Figs. 12, 13 and 14. Different gear transmission ratios are achieved by changing the number of gear teeth with the number of pinion teeth unchanged in Figs. 13 and 14.

Figure 12 shows that the module has little influence on the range of the inclination angle. The limit value of inclination angle changes greatly when the module is small, but the value is almost unchanged when the module is larger than 2 mm. The modulus has a great influence on the jet streamline offset. With the increase of the modulus, the maximum value of the jet streamline offset increases rapidly, while the minimum value decreases slightly. In addition, the modulus also affects the minimum oil jet velocity by influencing the pitch velocity.

Figure 13 shows the value range of oil jet inclination with the gear ratio when the jet streamline offset Δ*S* is 0.2 and 0 respectively. The value range of oil injection angle decreases with the increase of transmission ratio, but the change range is small, which indicates that the gear ratio has little effect on the inclination angle value during oil jet streamline positioning.

Figure 14 was prepared primarily to show the effect of gear ratio on the value range of jet streamline offset Δ*S* when *β* is 2.5°, 0° and − 2.5° respectively. At different inclination angles, the value range of jet streamline offset increases with the increase of gear ratio. The smaller the *β*, the greater the distance the jet streamline offset moves toward the pinion side. This is mainly due to the shielding effect of the gear on the pinion teeth becomes more and more obvious with the continuous increase of the gear ratio, resulting in the oil jet streamline unable to spray onto the pinion teeth. Therefore, to ensure that the gear pair are sprayed by lubricating oil, it is necessary to move the jet line toward the pinion side.

### Effect of initial offset

**Fig. 15:**
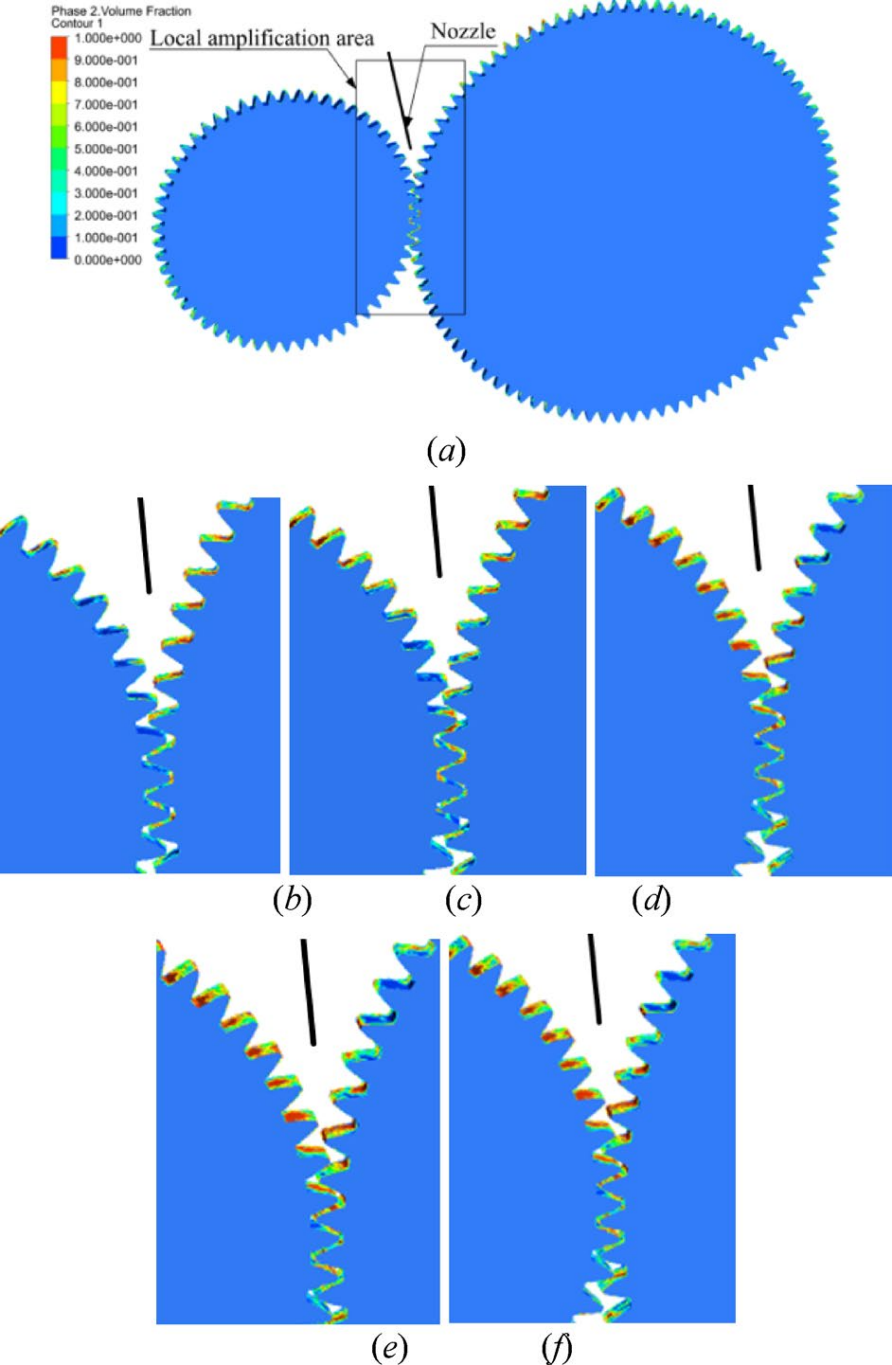
Oil volume fractions under different initial offset (**a**) global graph and partial enlarged details: (**b**) *S* = 0.2 mm; (**c)** *S* = 0.3 mm; (**d**) *S* = 0.4 mm; (**e**) *S* = 0.5 mm; (**f**) *S* = 0.6 mm.

According to the CFD simulation results of fluid field with inclination angle *β =* 4° and different initial offset in Sect. 4, the distribution of lubricating oil on tooth surface is obtained, as shown in Fig. 15.

With the increase of the initial offset *S*, the distribution of lubricating oil on the tooth surface of the pinion is more and more, while that of the gear is decreasing. Due to the high speed of the pinion in the gear pair, the friction and wear of the tooth surface is serious. In order to make the two gears fully lubricated and cooled, it can be seen from the distribution of the oil on the tooth surface that the oil spot area and oil volume fraction of tooth surface for the two gears are larger with the initial offset of 0.5 mm and 0.6 mm and the distribution in the tooth width direction is more uniform.

**Fig. 16:**
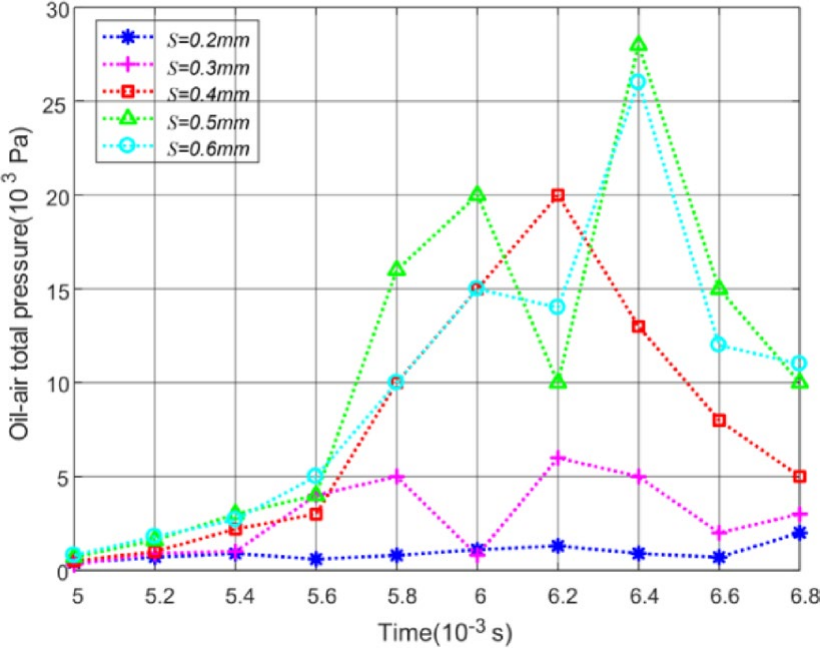
Effect of initial offset on oil pressure.

Based on the theory of Dowson^38,39^, the oil pressure on a plane near the meshing zone can be used as the criterion to judge the oil jet lubrication performance of gears. So, a bounded plane is made with coordinate Y = 0.1 mm near the meshing points. The instability data at the beginning of simulation calculation is ignored and the oil pressure distribution on this plane versus different initial offset at time 0.005s to 0.007s is obtained by CFD-POST, as shown in Fig. 16.

**Fig. 17:**
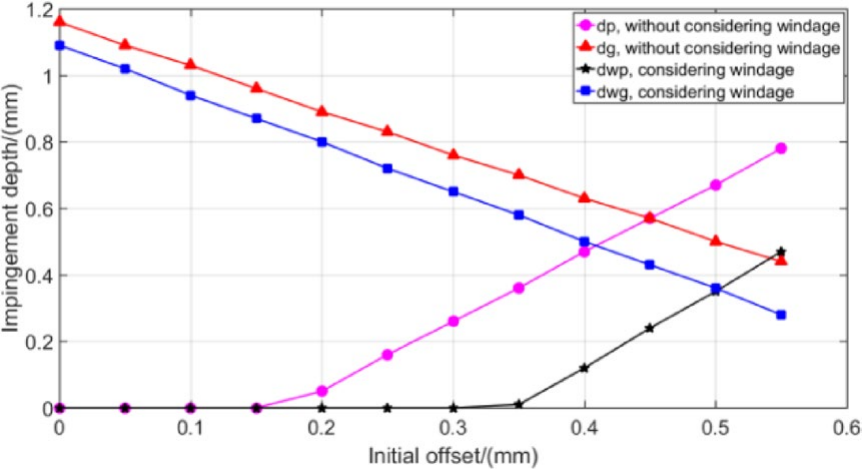
Effect of *S* on impingement depth.

As can be seen from Fig. 16, the oil pressure is the highest under the initial offset of 0.5 mm, which is consistent with the oil volume fraction distribution on the tooth surface. Figure 17 has been provided to give the reader a feel for the value of impingement depth obtained by theoretical model of Sect. 3 as a function of initial offset *S*.

Figure 17 shows that when parameter *S* is small, the oil jet streamline cannot reach the teeth of the pinion, and the impingement depth value of the gear is obviously greater than that of the pinion. However, with the continuous increase of *S*, the impingement depth value of the pinion increases rapidly, while the value of the gear decreases sharply. When *S* increases to a certain value, the impingement depth value of the pinion begins to gradually exceed the calculated value of the gear. The impingement depth of the two gears in the gear pair is negatively correlated. This indicates that parameter *S* has a great influence on the impingement depth value of gear pair, especially on the pinion. It also shows that *S* has a minimum value, that is, when the oil jet velocity is close to infinity, the jet streamline will miss the pinion. This is because when the gear is relatively large, the shielding effect of the gear teeth prevents the oil flow from spraying to the pinion teeth. At this time, it is necessary to set an appropriate larger *S* to ensure that both gear teeth can be fully lubricated and cooled. Figure 19 shows that the corresponding *S* value when the two curves of the pinion and gear intersect is the optimal *S* value, which is consistent with CFD simulation results.

### Effect of inclination angle

**Fig. 18:**
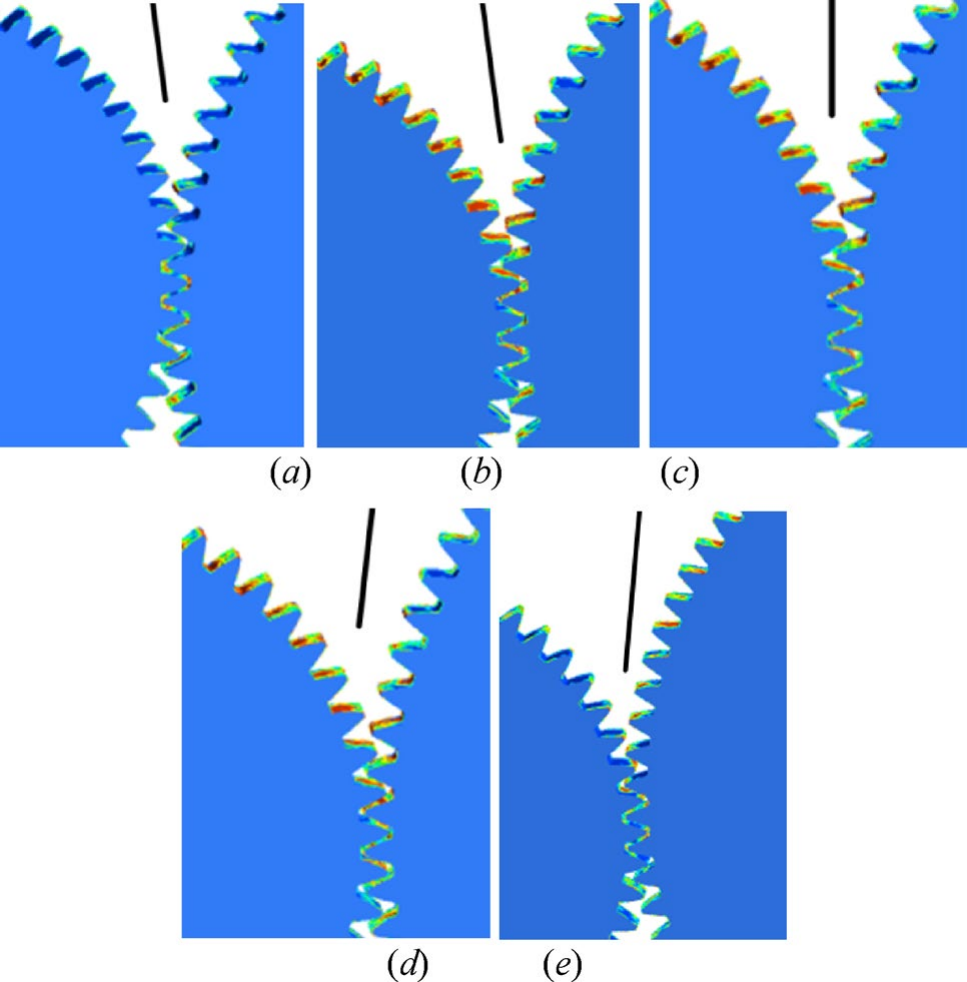
Oil volume fractions under different inclination angle (**a**) *β* = 10°; (**b**) *β* = 5°; (**c**) *β* = 0°; (**d**) *β*= -5°; (**e**) *β*= -10°.

The analysis method for the effect of inclination angle on oil volume fraction and oil pressure is the same as that in Sect. 6.2. Figure 18 is the partial enlarged details for the distribution of lubricating oil on tooth surface obtained from the CFD simulation results of fluid field with initial offset *S* = 0.5 mm and different inclination angles.

According to the lubricating oil distribution on tooth surface of the gear pair, we can know that the oil spot area is larger at the angle of 5°. Moreover, the lubricating oil is more evenly distributed along the tooth width, which indicates that the lubrication and cooling effect of the gear is better.

**Fig. 19:**
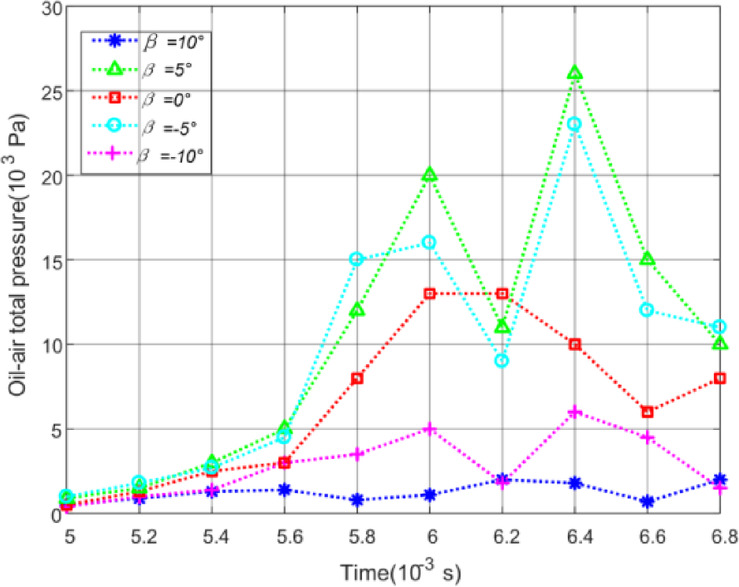
Effect of inclination angle *β* on oil pressure.

Figure 19 is the oil pressure distribution on the plane with coordinate Y = 0.1 mm near the meshing points versus different inclination angle at time 0.005s to 0.007s obtained by CFD-POST.

**Fig. 20:**
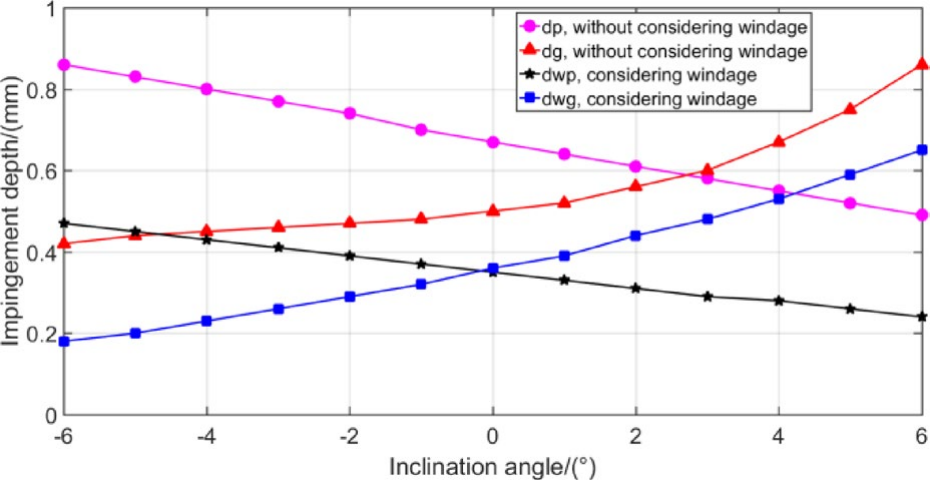
Effect of inclination angle *β* on impingement depth.

Figure 19 shows that the oil pressure reaches maximum value under the injection angle of 5°. And the excessive inclination angle obviously reduces the lubrication effect of both gears. By applying the mathematical model in Sect. 3 to calculate the oil jet impingement depth with different inclination angles, the corresponding impingement depth values can be calculated as shown in Fig. 20.

As shown in Fig. 20, with the increase of parameter *β*, the impingement depth of the pinion becomes smaller and even negative, while the impingement depth of the gear keeps increasing, and the impingement depth of the two gears is negatively correlated. Generally acknowledged, the pinion should be a prior and major concern as it is more susceptible to scuffing, pitting and scoring failure on tooth surface due to the poor heat dissipation capability for the pinion. Therefore, the nozzle should be inclined to the pinion, that is, the positive value is optimal. Combined with CFD simulation analysis, the injection angle should not be greater than 5°.

### Effect of oil jet velocity

**Fig. 21:**
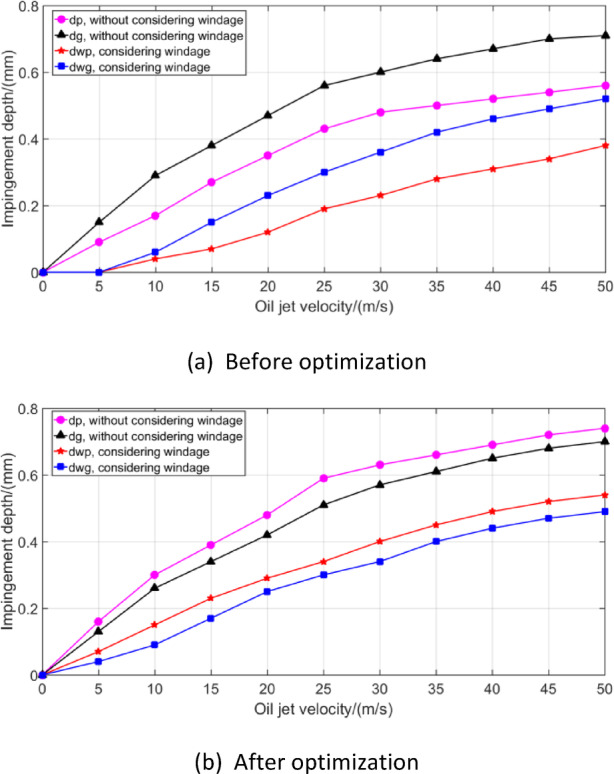
Effect of oil jet velocity on impingement depth.

The effect of oil jet velocity on impingement depth is shown in Fig. 21.

Figure 21a shows that the impingement depth values of the two gears are quite different at this time, and the value of the pinion is much smaller than that of the gear, especially when considering the windage effect. The impingement depth value of the pinion rapidly decreases to 0 with the decrease of oil jet velocity. That is, at this time, the pinion has a minimum oil jet velocity, which means that there must be sufficient oil pressure to provide the required oil jet velocity to ensure the possible maximum lubrication and cooling requirements. According to Fig. 21b, after optimizing the jet streamline layout parameters, the difference between the impingement depth values of the two gears is small, especially when considering the windage effect. In addition, it is nearly impossible to starve the gear teeth at any theoretically small jet velocity, but the impingement depth value is small. The impingement depth value increases with the increase of the oil jet velocity, which indicates that higher oil jet velocity could have more ability to resist the windage effect.

**Fig. 22:**
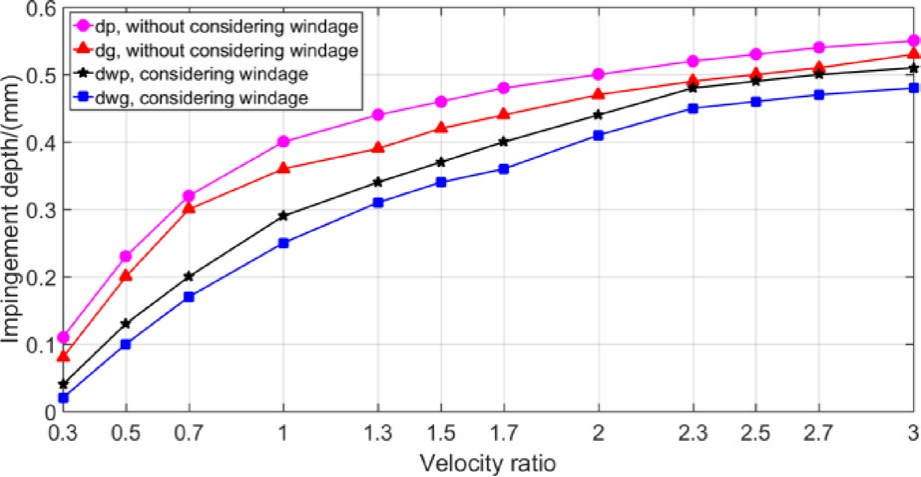
The impingement depth against different velocity ratio.

Figure 22 shows the change curve of impingement depth of gear pair with the ratio of oil jet velocity to pitch circle velocity after optimized design of jet streamline layout parameters. The windage impingement depth value increases with the increase of the velocity ratio, but the change rate gradually slows down. With the increase of gear speed, the surrounding air flow is accelerated, and the windage effect is enhanced. At this time, the windage will increase the air turbulence in the gearbox, causing the lubricating oil to be stirred, which may produce bubbles or oil mist, reducing the adhesion of the lubricating oil and the stability of the oil film^40^. At the same time, the windage effect may also cause uneven distribution of lubricating oil and insufficient oil in some areas, resulting in poor local lubrication and increasing the risk of wear.

Figure 22 shows that when the velocity ratio is greater than about 2.4, the growth rate of impingement depth of gear pair decreases. The maximum windage impingement depth value of the gear pair is about 0.5 mm, which is 9/100 of the tooth cavity depth, and this is consistent with the conclusion that the maximum impingement depth of the gear pair is about 1/10 of the tooth profile depth measured by Akin^21^ by experiments.

### Effect of droplet size

**Fig. 23:**
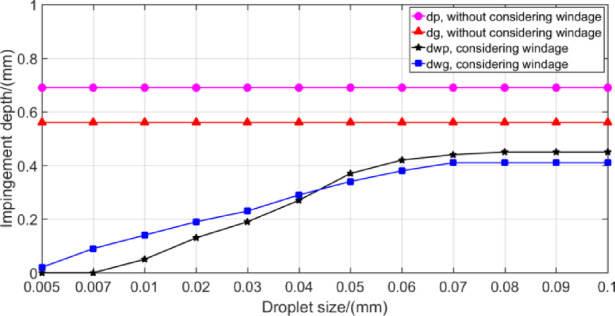
Effect of droplet size on impingement depth.

Figure 23 is prepared primarily to show the effect of drop size on the windage impingement depth. The 10,000 rpm rotation speed of the pinion and 25 m/s oil jet velocity are selected. It will be noticed that there is very little effect of droplet sizes larger than about 0.07 mm, but at droplet sizes below 0.01 mm, the effect of droplet size becomes very drastic, and the windage impingement depth value even becomes negative.

This indicates that when the oil droplet size in the oil jet streamline is small, it is difficult for the droplet to enter the meshing zone due to the influence of the windage, thus it is best not to choose the excessive atomization of the jet stream before impingement on the gear tooth.

## Conclusions

The calculation model of the windage impingement depth for aviation gear pair under oil jet lubrication is obtained.

The multi-objective NSGA-II intelligent optimization algorithm is used to optimize jet streamline layout parameters of the gear pair. The optimized nozzle layout parameters of the example in the article are *S* = 0.53 mm, *β* = 4.6°.

The results of CFD simulation analysis for the flow field of aviation high-speed gear pair validate the reliability and accuracy of the method proposed to evaluate the oil injection lubrication performance with windage impingement depth calculation model for aviation high-speed gear pair when considering windage effect.

The results show that: the module and gear ratio of gear pair mainly affect the value range of oil jet inclination angle and jet streamline offset; The inclination angle and the initial offset are negatively correlated with the impingement depth of the gear pair; The ratio of oil jet velocity to pitch circle velocity is the most important parameter affecting the oil jet lubrication of aviation high-speed gears; When the droplet size is less than 0.01 mm, the influence of windage on the impingement depth becomes larger and the contra-flow will happen.

## Data Availability

All data generated or analyzed during this study are included in this published article.
